# Supplementary material for: The Diagnostic Utility of Cell-Free DNA from Ex Vivo Bronchoalveolar Lavage Fluid in Lung Cancer
Source: Cancers (Basel). 2022 Mar 30;14(7):1764. doi: 10.3390/cancers14071764 (PMC8996852; doi:10.3390/cancers14071764)
Supplement: Supplementary file 1 [file cancers-14-01764-s001.zip › Supplementary Table S3.pdf]

Supplementary Table S3. Sequencing data in each sample.

Case No.	Sample	#_locus	type	ref	genotype	normal_genotype	gene	transcript	function	codon	exon	protein	coding		
1	FFPE	chr7:55242462	INDEL	CAAGGAATT<br>AAGAGAAGC	CAAGGAATT<br>AAGAGAAGC<br>/CAA	CAAGGAATT<br>AAGAGAAGC<br>/CAAGGAAT<br>TAAGAGAAG C	EGFR	NM_005228.4	nonframeshift<br>Deletion	-	19	p.Glu746_Ala<br>750del	c.2235_2249d<br>elGGAATTAA<br>GAGAAGC	*	#
		chrX:47045009	SNV	C	C/T	C/C	RBM10	NM_001204468.1	nonsense	TAG	20	p.Gln844Ter	c.2530C>T	*	#
		chr12:49443745	SNV	G	G/C	G/G	KMT2D	NM_003482.3	missense	TGT	11	p.Ser1209Cys	c.3626C>G		
	BAL	chr7:55242462	INDEL	CAAGGAATT<br>AAGAGAAGC	CAAGGAATT<br>AAGAGAAGC<br>/CAA	CAAGGAATT<br>AAGAGAAGC<br>/CAAGGAAT<br>TAAGAGAAG C	EGFR	NM_005228.4	nonframeshift<br>Deletion	-	19	p.Glu746_Ala<br>750del	c.2235_2249d<br>elGGAATTAA<br>GAGAAGC	*	#
		chrX:47045009	SNV	C	C/T	C/C	RBM10	NM_001204468.1	nonsense	TAG	20	p.Gln844Ter	c.2530C>T	*	#
2	FFPE	chr17:7577544	SNV	AT	AT/AA	AT/AT	TP53	NM_000546.5	missense	TTG	7	p.Met246Leu	c.736A>T	*	#
	BAL	chr17:7577545	SNV	T	T/A	T/T	TP53	NM_000546.5	missense	TTG	7	p.Met246Leu	c.736A>T	*	#
3	FFPE	chr7:55242462	INDEL	CAAGGAATT<br>AAGAGAAGC	CAAGGAATT<br>AAGAGAAGC<br>/CAA	CAAGGAATT<br>AAGAGAAGC<br>/CAAGGAAT<br>TAAGAGAAG C	EGFR	NM_005228.4	nonframeshift<br>Deletion	-	19	p.Glu746_Ala<br>750del	c.2235_2249d<br>elGGAATTAA<br>GAGAAGC	*	#
	BAL	chr7:55242464	INDEL	AGGAATTAA<br>GAGAAGC	AGGAATTAA<br>GAGAAGC/A	AGGAATTAA<br>GAGAAGC/A<br>GGAATTAAG<br>AGAAGC	EGFR	NM_005228.4	nonframeshift<br>Deletion	-	19	p.Glu746_Ala<br>750del	c.2235_2249d<br>elGGAATTAA<br>GAGAAGC	*	#
4	FFPE	chr12:25398280	SNV	GCCAC	GCCAC/GCC<br>AG	GCCAC/GCC AC	KRAS	NM_033360.3	missense	GCT	2	p.Gly12Ala	c.35G>C	*	#
		chr12:49448391	SNV	T	T/A	T/T	KMT2D	NM_003482.3	missense	GTG	3	p.Glu107Val	c.320A>T		
		chr15:42042316	SNV	A	A/T	A/A	MGA	NM_001164273.1	missense	TGG	17	p.Arg2171Trp	c.6511A>T		
		chrX:47045006	SNV	C	C/T	C/C	RBM10	NM_001204468.1	nonsense	TAG	20	p.Gln843Ter	c.2527C>T	*	
	BAL	chr9:139405209	SNV	C	C/T	C/C	NOTCH1	NM_017617.4	missense	CAG	17	p.Arg879Gln	c.2636G>A		
		chr12:25398280	SNV	GCCAC	GCCAC/GCC<br>AG	GCCAC/GCC AC	KRAS	NM_033360.3	missense	GCT	2	p.Gly12Ala	c.35G>C	*	#
		chr17:29653162	SNV	G	G/T	G/G	NF1	NM_001042492.2	missense	GAT	37	p.Glu1720Asp	c.5160G>T	*	
5	FFPE	chr16:3777869	SNV	G	G/C	G/G	CREBBP	NM_004380.2	missense	AGG	31	p.Ser2393Arg	c.7179C>G		
6	FFPE	chr7:55242462	INDEL	CAAGGAATT<br>AAGAGAAGC	CAAGGAATT<br>AAGAGAAGC<br>/CAA	CAAGGAATT<br>AAGAGAAGC<br>/CAAGGAAT<br>TAAGAGAAG C	EGFR	NM_005228.4	nonframeshift<br>Deletion	-	19	p.Glu746_Ala<br>750del	c.2235_2249d<br>elGGAATTAA<br>GAGAAGC	*	#
	BAL	chr7:55242462	INDEL	CAAGGAATT<br>AAGAGAAGC	CAAGGAATT<br>AAGAGAAGC<br>/CAA	CAAGGAATT<br>AAGAGAAGC<br>/CAAGGAAT<br>TAAGAGAAG C	EGFR	NM_005228.4	nonframeshift<br>Deletion	-	19	p.Glu746_Ala<br>750del	c.2235_2249d<br>elGGAATTAA<br>GAGAAGC	*	#
7	FFPE	chr2:178098800	SNV	T	T/A	T/T	NFE2L2	NM_006164.4	missense	GTA	2	p.Glu82Val	c.245A>T	*	#
		chr7:140500228	SNV	G	G/A	G/G	BRAF	NM_004333.4	missense	GTG	7	p.Ala305Val	c.914C>T	*	#
		chr17:7579358	SNV	C	C/A	C/C	TP53	NM_000546.5	missense	CTT	4	p.Arg110Leu	c.329G>T	*	#
	BAL	chr2:178098800	SNV	T	T/A	T/T	NFE2L2	NM_006164.4	missense	GTA	2	p.Glu82Val	c.245A>T	*	#
		chr7:140500228	SNV	G	G/A	G/G	BRAF	NM_004333.4	missense	GTG	7	p.Ala305Val	c.914C>T	*	#
		chr17:7579358	SNV	C	C/A	C/C	TP53	NM_000546.5	missense	CTT	4	p.Arg110Leu	c.329G>T	*	#
8	FFPE	chr3:178936091	SNV	G	G/C	G/G	PIK3CA	NM_006218.3	missense	CAG	10	p.Glu545Gln	c.1633G>C	*	
		chr7:55259515	SNV	TG	TG/GG	TG/TG	EGFR	NM_005228.4	missense	CGG	21	p.Leu858Arg	c.2573T>G	*	#
	BAL	chr7:55259515	SNV	TG	TG/GG	TG/TG	EGFR	NM_005228.4	missense	CGG	21	p.Leu858Arg	c.2573T>G	*	#
9	FFPE	chr19:1218491	MNV	GC	GC/TT	GC/GC	STK11	NM_000455.4	missense|non<br>sense	-	2	p.Lys122_Gln<br>123delinsAsn<br>Ter	c.366_367del<br>GCinsTT	*	#
		chr19:10602722	SNV	G	G/A	G/G	KEAP1	NM_203500.1	nonsense	TAG	3	p.Gln286Ter	c.856C>T	*	#
	BAL	chr19:1218491	MNV	GC	GC/TT	GC/GC	STK11	NM_000455.4	missense|non<br>sense	-	2	p.Lys122_Gln<br>123delinsAsn<br>Ter	c.366_367del<br>GCinsTT	*	#
		chr19:10602722	SNV	G	G/A	G/G	KEAP1	NM_203500.1	nonsense	TAG	3	p.Gln286Ter	c.856C>T	*	#
10	FFPE	chr1:27092857	MNV	GG	GG/TT	GG/GG	ARID1A	NM_006015.5	splice site	-	9	-	-	*	#
		chr2:178098944	SNV	C	C/T	C/C	NFE2L2	NM_006164.4	missense	CAA	2	p.Arg34Gln	c.101G>A	*	#
		chr2:225376224	SNV	C	C/T	C/C	CUL3	NM_003590.4	missense	AAA	6	p.Glu244Lys	c.730G>A		
		chr9:135771772	SNV	C	C/A	C/C	TSC1	NM_000368.4	missense	GAT	23	p.Glu1115Asp	c.3345G>T		#
		chr12:49426814	SNV	G	G/A	G/G	KMT2D	NM_003482.3	nonsense	TAG	39	p.Gln3892Ter	c.11674C>T	*	
		chr17:7579336	INDEL	CCCAGAATG<br>CAAGAAGCC<br>CAGACGGA<br>AA	CCCAGAATG<br>CAAGAAGCC<br>CAGACGGA<br>AA/C	CCCAGAATG<br>CAAGAAGCC<br>CAGACGGA<br>AA/CCCAGA<br>ATGCAAGAA<br>GCCCAGAC<br>GGAAA	TP53	NM_000546.5	nonframeshift<br>Deletion		4	p.Phe109_Gly<br>117del	c.324_350del<br>TTTCCGTCT<br>GGGCTTCT<br>TGCATTCTG<br>G		
		chr17:7579365	SNV	C	C/T	C/C	TP53	NM_000546.5	missense	AGT	4	p.Gly108Ser	c.322G>A		#
	BAL	chr1:27092857	MNV	GG	GG/TT	GG/GG	ARID1A	NM_006015.5	splice site	-	9	-	-	*	#
		chr2:178098944	SNV	C	C/T	C/C	NFE2L2	NM_006164.4	missense	CAA	2	p.Arg34Gln	c.101G>A	*	#
		chr9:135771772	SNV	C	C/A	C/C	TSC1	NM_000368.4	missense	GAT	23	p.Glu1115Asp	c.3345G>T		#
		chr17:7579365	SNV	C	C/T	C/C	TP53	NM_000546.5	missense	AGT	4	p.Gly108Ser	c.322G>A		#
11	FFPE	chr7:55259515	SNV	TG	TG/GG	TG/TG	EGFR	NM_005228.4	missense	CGG	21	p.Leu858Arg	c.2573T>G	*	#
	BAL	chr7:55259515	SNV	T	T/G	T/T	EGFR	NM_005228.4	missense	CGG	21	p.Leu858Arg	c.2573T>G	*	#
12	FFPE	chr3:47162156	SNV	G	G/A	G/G	SETD2	NM_014159.6	nonsense	TAA	3	p.Gln1324Ter	c.3970C>T	*	
13	FFPE	chr7:55242462	INDEL	CAAGGAATT<br>AAGAGAAGC	CAAGGAATT<br>AAGAGAAGC<br>/CAA	CAAGGAATT<br>AAGAGAAGC<br>/CAAGGAAT<br>TAAGAGAAG C	EGFR	NM_005228.4	nonframeshift<br>Deletion	-	19	p.Glu746_Ala<br>750del	c.2235_2249d<br>elGGAATTAA<br>GAGAAGC	*	#
		chr17:7577578	SNV	TGTA	TGTA/TGTG	TGTA/TGTA	TP53	NM_000546.5	missense	CAC	7	p.Tyr234His	c.700T>C	*	#
	BAL	chr7:55242462	INDEL	CAAGGAATT<br>AAGAGAAGC	CAAGGAATT<br>AAGAGAAGC<br>/CAA	CAAGGAATT<br>AAGAGAAGC<br>/CAAGGAAT<br>TAAGAGAAG C	EGFR	NM_005228.4	nonframeshift<br>Deletion	-	19	p.Glu746_Ala<br>750del	c.2235_2249d<br>elGGAATTAA<br>GAGAAGC	*	#
		chr17:7577578	SNV	TGTA	TGTA/TGTG	TGTA/TGTA	TP53	NM_000546.5	missense	CAC	7	p.Tyr234His	c.700T>C	*	#
14	FFPE	chr17:7579362	INDEL	AACCGT	AACCGT/AA<br>C	AACCGT/AA<br>CCGT	TP53	NM_000546.5	nonframeshift<br>Deletion	-	4	p.Tyr107_Gly<br>108delinsCys	c.320_322del<br>ACG	*	#
	BAL	chr17:7579362	INDEL	AACCGT	AACCGT/AA<br>C	AACCGT/AA<br>CCGT	TP53	NM_000546.5	nonframeshift<br>Deletion	-	4	p.Tyr107_Gly<br>108delinsCys	c.320_322del<br>ACG	*	#

15	FFPE	chr1:120458622	SNV	C	C/G	C/C	NOTCH2	NM_024408.3	missense	TTC	34	p.Leu2241Phe	c.6723G>C		#
		chr3:178952084	SNV	C	C/T	C/C	PIK3CA	NM_006218.3	missense	TAT	21	p.His1047Tyr	c.3139C>T	*	#
		chr12:49427229	SNV	G	G/C	G/G	KMT2D	NM_003482.3	missense	ATG	39	p.Ile3753Met	c.11259C>G		
		chr17:7577528	SNV	GATGGGCCTCCGGTTC	GATGGGCCTCCGGTTC/GATGGGCCTCAGGTTC	GATGGGCCTCCGGTTC/GATGGGCCTCCGGTTC	TP53	NM_000546.5	missense	CTG	7	p.Arg248Leu	c.743G>T	*	#
		chr17:29527558	SNV	G	G/T	G/G	NF1	NM_001042492.2	missense	TTG	9	p.Trp336Leu	c.1007G>T		#
	BAL	chr1:120458622	SNV	C	C/G	C/C	NOTCH2	NM_024408.3	missense	TTC	34	p.Leu2241Phe	c.6723G>C		#
		chr3:178952084	SNV	C	C/T	C/C	PIK3CA	NM_006218.3	missense	TAT	21	p.His1047Tyr	c.3139C>T	*	#
		chr17:7577528	SNV	GATGGGCCTCCGGTTC	GATGGGCCTCCGGTTC/GATGGGCCTCAGGTTC	GATGGGCCTCCGGTTC/GATGGGCCTCCGGTTC	TP53	NM_000546.5	missense	CTG	7	p.Arg248Leu	c.743G>T	*	#
		chr17:29527558	SNV	G	G/T	G/G	NF1	NM_001042492.2	missense	TTG	9	p.Trp336Leu	c.1007G>T		#
		chr19:11152197	SNV	T	T/G	T/T	SMARCA4	NM_001128849.1	missense	AGG	31	p.Met1494Arg	c.4481T>G		
16	FFPE	chr7:55259515	SNV	TG	TG/GG	TG/TG	EGFR	NM_005228.4	missense	CGG	21	p.Leu858Arg	c.2573T>G	*	#
		chr11:533499	SNV	C	C/T	C/C	HRAS	NM_001130442.2	missense	CAA	4	p.Arg135Gln	c.404G>A	*	
		chr11:108121704	SNV	C	C/A	C/C	ATM	NM_000051.3	missense	AAA	10	p.Asn504Lys	c.1512C>A		
		chrX:47039818	SNV	G	G/C	G/G	RBM10	NM_001204468.1	missense	AGC	12	p.Arg452Ser	c.1356G>C		#
	BAL	chr7:55259515	SNV	TG	TG/GG	TG/TG	EGFR	NM_005228.4	missense	CGG	21	p.Leu858Arg	c.2573T>G	*	#
		chrX:47039818	SNV	G	G/C	G/G	RBM10	NM_001204468.1	missense	AGC	12	p.Arg452Ser	c.1356G>C		#
17	FFPE	chr7:55259515	SNV	TG	TG/GG	TG/TG	EGFR	NM_005228.4	missense	CGG	21	p.Leu858Arg	c.2573T>G	*	#
		chr17:7577070	SNV	GGAGATTCTCTTCCTCTGTGC	GGAGATTCTCTTCCTCTGTGC/GGAGATTCTCTTCCTTTGTGC	GGAGATTCTCTTCCTCTGTGC/GGAGATTCTCTTCCTCTGTGC	TP53	NM_000546.5	missense	AAG	8	p.Glu285Lys	c.853G>A	*	#
	BAL	chr7:55259515	SNV	TG	TG/GG	TG/TG	EGFR	NM_005228.4	missense	CGG	21	p.Leu858Arg	c.2573T>G	*	#
		chr17:7577070	SNV	GGAGATTCTCTTCCTCTGTGC	GGAGATTCTCTTCCTCTGTGC/GGAGATTCTCTTCCTTTGTGC	GGAGATTCTCTTCCTCTGTGC/GGAGATTCTCTTCCTCTGTGC	TP53	NM_000546.5	missense	AAG	8	p.Glu285Lys	c.853G>A	*	#
18	FFPE	chr3:47079256	SNV	C	C/A	C/C	SETD2	NM_014159.6	missense	TTG	18	p.Trp2417Leu	c.7250G>T		
		chr4:20598068	SNV	G	G/T	G/G	SLIT2	NM_004787.3	missense	ATT	32	p.Met1117Ile	c.3351G>T		
		chr12:46244334	SNV	G	G/T	G/G	ARID2	NM_152641.3	missense	TCA	15	p.Ala810Ser	c.2428G>T		#
		chr12:49425545	SNV	G	G/A	G/G	KMT2D	NM_003482.3	nonsense	TAA	39	p.Gln4315Ter	c.12943C>T	*	
		chr17:7577142	SNV	C	C/A	C/C	TP53	NM_000546.5	nonsense	TGA	8	p.Gly266Ter	c.796G>T	*	#
		chr19:11098380	SNV	C	C/T	C/C	SMARCA4	NM_001128849.1	nonsense	TAG	6	p.Gln300Ter	c.898C>T	*	
		chrX:47030526	SNV	G	G/T	G/G	RBM10	NM_001204468.1	missense	TAC	4	p.Asp166Tyr	c.496G>T	*	
	BAL	chr12:46244334	SNV	G	G/T	G/G	ARID2	NM_152641.3	missense	TCA	15	p.Ala810Ser	c.2428G>T		#
		chr17:7577142	SNV	C	C/A	C/C	TP53	NM_000546.5	nonsense	TGA	8	p.Gly266Ter	c.796G>T	*	#
19	FFPE	chr7:55259515	SNV	TG	TG/GG	TG/TG	EGFR	NM_005228.4	missense	CGG	21	p.Leu858Arg	c.2573T>G	*	
		chr10:89692818	SNV	T	T/C	T/T	PTEN	NM_000314.6	missense	ACC	5	p.Ile101Thr	c.302T>C	*	#
		chrX:47030535	SNV	G	G/A	G/G	RBM10	NM_001204468.1	missense	AAC	4	p.Asp169Asn	c.505G>A		
	BAL	chr10:89692818	SNV	T	T/C	T/T	PTEN	NM_000314.6	missense	ACC	5	p.Ile101Thr	c.302T>C	*	#
20	FFPE	chr7:55259515	SNV	TG	TG/GG	TG/TG	EGFR	NM_005228.4	missense	CGG	21	p.Leu858Arg	c.2573T>G	*	#
		chr17:7577104	SNV	AGG	AGG/AGA	AGG/AGG	TP53	NM_000546.5	missense	TCT	8	p.Pro278Ser	c.832C>T	*	#
		chr17:7577559	SNV	GA	GA/AA	GA/GA	TP53	NM_000546.5	missense	TTC	7	p.Ser241Phe	c.722C>T	*	
		chr17:7578181	SNV	G	G/C	G/G	TP53	NM_000546.5	missense	CGT	6	p.Pro223Arg	c.668C>G	*	
		chrX:47045136	SNV	C	C/T	C/C	RBM10	NM_001204468.1	nonsense	TGA	21	p.Arg858Ter	c.2572C>T	*	
	BAL	chr7:55259515	SNV	TG	TG/GG	TG/TG	EGFR	NM_005228.4	missense	CGG	21	p.Leu858Arg	c.2573T>G	*	#
		chr17:7577104	SNV	AGG	AGG/AGA	AGG/AGG	TP53	NM_000546.5	missense	TCT	8	p.Pro278Ser	c.832C>T	*	#
21	FFPE	chr6:157099449	INDEL	A	A/AGCA	A/A	ARID1B	NM_020732.3	nonframeshiftInsertion	-	1	p.Gln129dup	c.386_387insGCA		
		chr7:55242462	INDEL	CAAGGAATTAAGAGAAGCA	CAAGGAATTAAGAGAAGCA/CAAG	CAAGGAATTAAGAGAAGCA/CAAGGAATTAAGAGAAGCA	EGFR	NM_005228.4	nonframeshift Deletion	-	19	p.Glu746_Ala750del	c.2236_2250delGAATTAAGAGAAGCA	*	#
		chr17:7578368	SNV	CACCATCGCTATCTGAGCAGCGCTCATGGTGGGGGCAGCGCCTCACA	CACCATCGCTATCTGAGCAGCGCTCATGGTGGGGGCAGCGCCTCACA/CACCATCGCTATCTGAGCAGCGCTCACGGTGGGGGCAGCGCCTCACA	CACCATCGCTATCTGAGCAGCGCTCATGGTGGGGGCAGCGCCTCACA/CACCATCGCTATCTGAGCAGCGCTCATGGTGGGGGCAGCGCCTCACA	TP53	NM_000546.5	missense	CGT	5	p.His179Arg	c.536A>G	*	
	BAL	chr7:55242462	INDEL	CAAGGAATTAAGAGAAGCA	CAAGGAATTAAGAGAAGCA/CAAG	CAAGGAATTAAGAGAAGCA/CAAGGAATTAAGAGAAGCA	EGFR	NM_005228.4	nonframeshift Deletion	-	19	p.Glu746_Ala750del	c.2236_2250delGAATTAAGAGAAGCA	*	#
22	FFPE	chr11:118365088	SNV	G	G/C	G/G	KMT2A	NM_001197104.1	missense	ACC	17	p.Ser1755Thr	c.5264G>C		#
		chr12:49420349	INDEL	GAC	GAC/G	GAC/GAC	KMT2D	NM_003482.3	frameshiftDeletion	TCC	48	p.Cys5133fs	c.15398_15399delGT	*	#
		chr17:29657381	SNV	C	C/A	C/C	NF1	NM_001042492.2	missense	ATA	39	p.Leu1893Ile	c.5677C>A		#
	BAL	chr11:118365088	SNV	G	G/C	G/G	KMT2A	NM_001197104.1	missense	ACC	17	p.Ser1755Thr	c.5264G>C		#
		chr12:49420349	INDEL	GAC	GAC/G	GAC/GAC	KMT2D	NM_003482.3	frameshiftDeletion	TCC	48	p.Cys5133fs	c.15398_15399delGT	*	#
		chr17:29657381	SNV	C	C/A	C/C	NF1	NM_001042492.2	missense	ATA	39	p.Leu1893Ile	c.5677C>A		#
23	FFPE	chr17:7578452	SNV	TGGCGCG	TGGCGCG/TGGGGCG	TGGCGCG/TGGCGCG	TP53	NM_000546.5	missense	CCC	5	p.Ala159Pro	c.475G>C	*	#
		chr17:7578454	INDEL	GC	GC/G	GC/GC	TP53	NM_000546.5	frameshiftDeletion	CCA	5	p.Ala159fs	c.475delG	*	#
		chr9:21971208	SNV	C	C/G	C/C	CDKN2A	NM_001195132.1	splice site	-	2	-	c.151-1G>C	*	#
	BAL	chr17:7578452	SNV	TGGCGCG	TGGCGCG/TGGGGCG	TGGCGCG/TGGCGCG	TP53	NM_000546.5	missense	CCC	5	p.Ala159Pro	c.475G>C	*	#
		chr17:7578454	INDEL	GC	GC/G	GC/GC	TP53	NM_000546.5	frameshiftDeletion	CCA	5	p.Ala159fs	c.475delG	*	#
		chr9:21971208	SNV	C	C/G	C/C	CDKN2A	NM_001195132.1	splice site	-	2	-	c.151-1G>C	*	#
24	FFPE	chr7:55241678	INDEL	AAAC	AAAC/A	AAAC/AAAC	EGFR	NM_005228.4	nonframeshift Deletion	-	18	p.Glu709_Thr710delinsAsp	c.2127_2129delAAC		
		chr17:7578211	SNV	CG	CG/AG	CG/CG	TP53	NM_000546.5	missense	CTA	6	p.Arg213Leu	c.638G>T	*	#
	BAL	chr17:7578211	SNV	CG	CG/AG	CG/CG	TP53	NM_000546.5	missense	CTA	6	p.Arg213Leu	c.638G>T	*	#
25	FFPE	chr17:7578534	SNV	CTTG	CTTG/CTCG	CTTG/CTTG	TP53	NM_000546.5	missense	GAG	5	p.Lys132Glu	c.394A>G	*	#
	BAL	chr1:27057873	SNV	T	T/A	T/T	ARID1A	NM_006015.5	missense	CAA	3	p.His527Gln	c.1581T>A		
		chr11:108224588	SNV	G	G/C	G/G	ATM	NM_000051.3	missense	CTT	60	p.Val2923Leu	c.8767G>C		
		chr15:42059456	SNV	C	C/T	C/C	MGA	NM_001164273.1	missense	TTA	24	p.Ser3059Leu	c.9176C>T		
		chr15:66727514	SNV	G	G/C	G/G	MAP2K1	NM_002755.3	missense	GCC	2	p.Gly77Ala	c.230G>C		
		chr17:7578534	SNV	CTTG	CTTG/CTCG	CTTG/CTTG	TP53	NM_000546.5	missense	GAG	5	p.Lys132Glu	c.394A>G	*	#

26	FFPE	chr1:27087940	SNV	C	C/G	C/C	ARID1A	NM_006015.5	missense	GAA	6	p.Gln743Glu	c.2227C>G		
		chr2:178098810	SNV	C	C/T	C/C	NFE2L2	NM_006164.4	missense	AAG	2	p.Glu79Lys	c.235G>A	*	
		chr3:47163035	SNV	C	C/T	C/C	SETD2	NM_014159.6	missense	ATG	3	p.Val1031Met	c.3091G>A		#
		chr3:178936091	SNV	G	G/A	G/G	PIK3CA	NM_006218.3	missense	AAG	10	p.Glu545Lys	c.1633G>A	*	#
		chr5:86672277	SNV	C	C/G	C/C	RASA1	NM_002890.2	missense	AGG	16	p.Ser693Arg	c.2079C>G		#
		chr7:51096248	SNV	C	C/T	C/C	COBL	NM_015198.4	missense	ACT	10	p.Ala849Thr	c.2545G>A		
		chr12:49431850	SNV	C	C/T	C/C	KMT2D	NM_003482.3	missense	AAG	34	p.Glu3097Lys	c.9289G>A		#
	BAL	chr3:47163035	SNV	C	C/T	C/C	SETD2	NM_014159.6	missense	ATG	3	p.Val1031Met	c.3091G>A		#
		chr3:178936091	SNV	G	G/A	G/G	PIK3CA	NM_006218.3	missense	AAG	10	p.Glu545Lys	c.1633G>A	*	#
		chr5:86672277	SNV	C	C/G	C/C	RASA1	NM_002890.2	missense	AGG	16	p.Ser693Arg	c.2079C>G		#
		chr12:49431850	SNV	C	C/T	C/C	KMT2D	NM_003482.3	missense	AAG	34	p.Glu3097Lys	c.9289G>A		#
27	FFPE	chr7:116412043	SNV	G	G/A	G/G	MET	NM_001127500.2	missense	AAT	14	p.Asp1028Asn	c.3082G>A	*	#
		chr12:49446025	SNV	C	C/T	C/C	KMT2D	NM_003482.3	missense	AAG	10	p.Glu481Lys	c.1441G>A		#
		chr13:49030428	SNV	G	G/A	G/G	RB1	NM_000321.2	missense	ACC	19	p.Ala635Thr	c.1903G>A		
	BAL	chr7:116412043	SNV	G	G/A	G/G	MET	NM_001127500.2	missense	AAT	14	p.Asp1028Asn	c.3082G>A	*	#
		chr12:49446025	SNV	C	C/T	C/C	KMT2D	NM_003482.3	missense	AAG	10	p.Glu481Lys	c.1441G>A		#
28	FFPE	chr4:1808587	SNV	G	G/A	G/G	FGFR3	NM_000142.4	missense	ACG	17	p.Ala734Thr	c.2200G>A		#
		chr5:86564321	SNV	C	C/G	C/C	RASA1	NM_002890.2	missense	GGT	1	p.Ala18Gly	c.53C>G		#
		chr7:55242462	INDEL	CAAGGAATTAAGAGAAGCAACATCT	CAAGGAATTAAGAGAAGCAACATCT/CAAGGAA	CAAGGAATTAAGAGAAGCAACATCT/CAAGGAATTAAGAGAAGCAACATCT	EGFR	NM_005228.4	nonframeshift Deletion		19	p.Leu747_Ser752del	c.2239_2256delTTAAGAGAAGCAACATCT	*	#
		chr7:55242491	SNV	A	A/G	A/A	EGFR	NM_005228.4	missense	AGA	19	p.Lys754Arg	c.2261A>G	*	#
		chr7:55242493	SNV	G	G/T	G/G	EGFR	NM_005228.4	missense	TCC	19	p.Ala755Ser	c.2263G>T		#
		chr13:49030428	SNV	G	G/A	G/G	RB1	NM_000321.2	missense	ACC	19	p.Ala635Thr	c.1903G>A	*	
		chr17:7578368	SNV	CACCATCGCTATCTGAGCAGCGCTCATGGTGGGGGCAGCGCCTCACA	CACCATCGCTATCTGAGCAGCGCTCATGGTGGGGGCAGCGCCTCACA/CACCATCGCTATCTCAGCAGCGCTCATGGTGGGGGCAGCGCCTCACA	CACCATCGCTATCTGAGCAGCGCTCATGGTGGGGGCAGCGCCTCACA/CACCATCGCTATCTGAGCAGCGCTCATGGTGGGGGCAGCGCCTCACA	TP53	NM_000546.5	nonsense	TGA	5	p.Ser183Ter	c.548C>G	*	#
		chr15:42021492	SNV	C	C/G	C/C	MGA	NM_001164273.1	nonsense	TGA	11	p.Ser1263Ter	c.3788C>G	*	#
	BAL	chr4:1808587	SNV	G	G/A	G/G	FGFR3	NM_000142.4	missense	ACG	17	p.Ala734Thr	c.2200G>A		#
		chr5:86564321	SNV	C	C/G	C/C	RASA1	NM_002890.2	missense	GGT	1	p.Ala18Gly	c.53C>G		#
		chr7:55242462	INDEL	CAAGGAATTAAGAGAAGCAACATCT	CAAGGAATTAAGAGAAGCAACATCT/CAAGGAA	CAAGGAATTAAGAGAAGCAACATCT/CAAGGAATTAAGAGAAGCAACATCT	EGFR	NM_005228.4	nonframeshift Deletion		19	p.Leu747_Ser752del	c.2239_2256delTTAAGAGAAGCAACATCT	*	#
		chr7:55242491	SNV	A	A/G	A/A	EGFR	NM_005228.4	missense	AGA	19	p.Lys754Arg	c.2261A>G	*	#
		chr7:55242493	SNV	G	G/T	G/G	EGFR	NM_005228.4	missense	TCC	19	p.Ala755Ser	c.2263G>T		#
		chr15:42021492	SNV	C	C/G	C/C	MGA	NM_001164273.1	nonsense	TGA	11	p.Ser1263Ter	c.3788C>G	*	#
		chr17:7578368	SNV	CACCATCGCTATCTGAGCAGCGCTCATGGTGGGGGCAGCGCCTCACA	CACCATCGCTATCTGAGCAGCGCTCATGGTGGGGGCAGCGCCTCACA/CACCATCGCTATCTCAGCAGCGCTCATGGTGGGGGCAGCGCCTCACA	CACCATCGCTATCTGAGCAGCGCTCATGGTGGGGGCAGCGCCTCACA/CACCATCGCTATCTGAGCAGCGCTCATGGTGGGGGCAGCGCCTCACA	TP53	NM_000546.5	nonsense	TGA	5	p.Ser183Ter	c.548C>G	*	#
29	FFPE	chr12:25398280	SNV	GCCACC	GCCACC/GCCACA	GCCACC/GCCACC	KRAS	NM_033360.3	missense	TGT	2	p.Gly12Cys	c.34G>T	*	#
		chrX:47041244	SNV	C	C/T	C/C	RBM10	NM_001204468.1	nonsense	TAG	15	p.Gln623Ter	c.1867C>T	*	#
	BAL	chr12:25398280	SNV	GCCACC	GCCACC/GCCACA	GCCACC/GCCACC	KRAS	NM_033360.3	missense	TGT	2	p.Gly12Cys	c.34G>T	*	#
		chrX:47041244	SNV	C	C/T	C/C	RBM10	NM_001204468.1	nonsense	TAG	15	p.Gln623Ter	c.1867C>T	*	#
30	FFPE	chr22:41574707	SNV	T	T/C	T/T	EP300	NM_001429.3	missense	ACG	31	p.Met2331Thr	c.6992T>C		#
	BAL	chr22:41574707	SNV	T	T/C	T/T	EP300	NM_001429.3	missense	ACG	31	p.Met2331Thr	c.6992T>C		#
31	FFPE	chr7:55259515	SNV	TG	TG/GG	TG/TG	EGFR	NM_005228.4	missense	CGG	21	p.Leu858Arg	c.2573T>G	*	#
	BAL	chr7:55259515	SNV	TG	TG/GG	TG/TG	EGFR	NM_005228.4	missense	CGG	21	p.Leu858Arg	c.2573T>G	*	#
32	FFPE	chr17:7578211	SNV	CG	CG/CA	CG/CG	TP53	NM_000546.5	nonsense	TGA	6	p.Arg213Ter	c.637C>T	*	#
		chr17:29670147	INDEL	TTA	TTA/T	TTA/TTA	NF1	NM_001042492.2	nonsense	TAA	48	p.Leu2395Ter	c.7184_7185delTA	*	#
	BAL	chr17:7578211	SNV	CG	CG/CA	CG/CG	TP53	NM_000546.5	nonsense	TGA	6	p.Arg213Ter	c.637C>T	*	#
		chr17:29670147	INDEL	TTA	TTA/T	TTA/TTA	NF1	NM_001042492.2	nonsense	TAA	48	p.Leu2395Ter	c.7184_7185delTA	*	#

*, oncogenic mutation; #, shared mutation between primary lesion and BALF.
