# Supplementary material for: The Diagnostic Utility of Cell-Free DNA from Ex Vivo Bronchoalveolar Lavage Fluid in Lung Cancer
Source: Cancers (Basel). 2022 Mar 30;14(7):1764. doi: 10.3390/cancers14071764 (PMC8996852; doi:10.3390/cancers14071764)
Supplement: Supplementary file 1 [file cancers-14-01764-s001.zip › SuppleTable S1.pdf]

Supplementary Table S1. The genes targeted in the cancer panel.

No	Gene symbol	Chromosome	Number of Amplicons	Total Bases	Covered Bases	Overall Coverage
1	*AKT1*	chr14	26	1573	1497	95%
2	*AKT2*	chr19	27	1576	1543	98%
3	*AKT3*	chr1	30	1624	1624	100%
4	*ARID1A*	chr1	76	7058	6023	85%
5	*ARID1B*	chr6	75	6950	5965	86%
6	*ARID2*	chr12	71	5718	5643	99%
7	*ASCL4*	chr12	5	532	382	72%
8	*ATM*	chr11	147	9791	9439	96%
9	*BRAF*	chr7	37	2481	2224	90%
10	*CDKN2A*	chr9	9	962	612	64%
11	*COBL*	chr7	48	4151	3977	96%
12	*CREBBP*	chr16	96	7639	7071	93%
13	*CTNNB1*	chr3	32	2486	2486	100%
14	*CUL3*	chr2	42	2561	2495	97%
15	*EGFR*	chr7	60	4189	4135	99%
16	*EP300*	chr22	90	7555	7182	95%
17	*EPHA7*	chr6	44	3175	3154	99%
18	*ERBB2*	chr17	57	4080	3808	93%
19	*ERBB3*	chr12	59	4440	4374	99%
20	*FGFR1*	chr8	41	2825	2816	100%
21	*FGFR2*	chr10	43	2910	2842	98%
22	*FGFR3*	chr4	34	2752	2215	81%
23	*FOXP2*	chr7	36	2487	2469	99%
24	*HRAS*	chr11	11	683	683	100%
25	*KEAP1*	chr19	24	1925	1845	96%
26	*KMT2D*	chr12	192	17154	15854	92%
27	*KRAS*	chr12	10	737	681	92%
28	*MAP2K1*	chr15	18	1292	1239	96%
29	*MET*	chr7	59	4427	4396	99%
30	*MGA*	chr15	110	9428	9345	99%
31	*MLL*	chr11	144	12279	11875	97%
32	*NF1*	chr17	136	9161	9023	99%
33	*NFE2L2*	chr2	23	1868	1826	98%
34	*NOTCH1*	chr9	99	8008	7078	88%
35	*NOTCH2*	chr1	101	7809	7539	97%
36	*NRAS*	chr1	9	610	610	100%
37	*PIK3CA*	chr3	50	3407	3282	96%
38	*PTEN*	chr10	18	1302	1223	94%
39	*RASA1*	chr5	55	3412	3216	94%
40	*RB1*	chr13	55	3057	2902	95%
41	*RBM10*	chrX	48	3228	3079	95%
42	*RIT1*	chr1	13	771	771	100%
43	*SETD2*	chr3	91	7905	7663	97%
44	*SLIT2*	chr4	76	4972	4854	98%
45	*SMAD4*	chr18	24	1769	1715	97%
46	*SMARCA4*	chr19	74	5399	5055	94%
47	*SOX2*	chr3	9	964	883	92%
48	*STK11*	chr19	23	1392	1343	97%
49	*TP53*	chr17	22	1383	1351	98%
50	*TP63*	chr3	34	2360	2227	94%
51	*TSC1*	chr9	49	3705	3603	97%
52	*TSC2*	chr16	92	5834	5677	97%
53	*U2AF1*	chr21	15	880	870	99%
