# Supplementary material for: The Diagnostic Utility of Cell-Free DNA from Ex Vivo Bronchoalveolar Lavage Fluid in Lung Cancer
Source: Cancers (Basel). 2022 Mar 30;14(7):1764. doi: 10.3390/cancers14071764 (PMC8996852; doi:10.3390/cancers14071764)
Supplement: Supplementary file 1 [file cancers-14-01764-s001.zip › SuppleTable S2.pdf]

Supplementary Table S2. Patient characteristics.

Case No.	age	sex	smoking habit	operative procedure	size (cm)	Histopathology	Histopathology sub	T	N	M	p-stage	BAL cytology	BAL sup. Genome Dx	BAL ppt. Genome Dx	ct DNA Genome Dx
1	53	female	+	right upper lobectomy	3.5	ad	papillary predominant	2a	2	0	IIIA	I	+	+	-
2	74	male	+	right upper lobectomy	3	ad	solid predominant	2a	2	0	IIIA	I	+	-	+
3	66	female	-	right lower lobectomy	1.3	ad	MIA	1mi	0	0	IA	I	-	+	-
4	87	male	+	right lower lobectomy	2.5	ad	lepidic predominant	1b	0	0	IA	II	+	-	-
5	54	female	-	right upper lobectomy	2.3	ad	lepidic predominant	1a	0	0	IA	I	-	-	-
6	60	female	+	right upper lobectomy	4	ad	lepidic predominant	1a	0	0	IA	I	-	+	-
7	59	male	+	right upper lobectomy	4.3	sq	moderately differentiated	2a	2	0	IIIA	I	+	-	-
8	79	male	+	right lower lobectomy	3.5	ad	papillary predominant	2a	0	0	IB	I	+	+	-
9	76	male	+	left upper lobectomy	3.7	pleomorphic		2a	0	0	IB	I	+	+	-
10	86	male	+	right lower lobectomy	3	sq	poorly differentiated	1c	0	0	IA	I	+	-	-
11	69	female	-	right upper lobectomy	2.3	ad	lepidic predominant	2a	0	0	IB	I	+	+	-
12	74	female	-	right upper lobectomy	3.1	ad	MIA	1mi	0	0	IA	I	-	-	-
13	56	female	-	right lower lobectomy	2.3	ad	acinar predominant	2a	0	0	IB	I	+	-	-
14	56	female	-	left upper lobectomy	4.5	ad	acinar predominant	2a	0	0	IB	I	+	-	-
15	57	male	+	left upper lobectomy	5.4	pleomorphic		3	0	0	IIB	I	+	-	-
16	81	female	-	right upper lobectomy	3.9	ad	lepidic predominant	1c	0	0	IA	I	+	+	-
17	72	female	-	right lower lobectomy	2.2	ad	acinar predominant	1b	0	0	IA	I	+	-	-
18	62	female	+	right upper lobectomy	1.8	ad	solid predominant	2a	0	0	IB	I	+	-	-
19	68	female	-	right upper lobectomy	3	ad	acinar predominant	1b	0	0	IA	I	+	-	
20	73	female	-	left upper lobectomy	2.8	ad	papillary predominant	2a	1	0	IIB	I	+	-	-
21	84	female	-	right lower lobectomy	2.1	ad	lepidic predominant	1a	0	0	IA	I	-	+	-
22	72	female	+	right lower lobectomy	2.1	sq	well to moderately differentiated	2a	0	0	IB	IV	+	+	-
23	67	female	+	right upper lobectomy	2.9	sq	moderately differentiated	1c	0	0	IA	I	+	+	-
24	49	male	+	left upper lobectomy	2.5	ad	lepidic predominant	1b	0	0	IA	I	+	-	-
25	70	female	+	left upper lobectomy	2.5	sq	poorly differntiated	1c	0	0	IA	I	+	-	-
26	56	male	+	left lower lobectomy	1.5	sq	keratinaizing, moderately differntiated	2a	0	0	IB	II	+	-	-
27	87	female	+	right upper lobectomy	5.4	adsq		2a	0	0	IB	I	+	+	-
28	69	male	+	left lower lobectomy	2.3	ad	acinar predominant	1b	0	0	IA	I	+	+	-
29	69	male	+	right upper lobectomy	2	ad	lepidic predominant	1a	0	0	IA	I	+	-	-
30	78	female	-	left upper lobectomy	4.7	LCNEC		2a	0	1b	IV	I	-	+	-
31	88	female	-	left lower lobectomy	2.3	ad	lepidic predominant	1b	0	0	IA	I	+	-	-
32	83	male	+	right lower lobectomy	2.6	ad	papillary predominant	2b	0	0	IIA	I	-	+	-

Dx, diagnosis; ad, adenocarcinoma; sq, squamous cell carcinoma; adsq, adenosquamous carcinoma: LCNEC, large cell neuroendocrine carcinoma; MIA, minimally invasive adenocarcinoma.
