# Supplementary material for: The Diagnostic Utility of Cell-Free DNA from Ex Vivo Bronchoalveolar Lavage Fluid in Lung Cancer
Source: Cancers (Basel). 2022 Mar 30;14(7):1764. doi: 10.3390/cancers14071764 (PMC8996852; doi:10.3390/cancers14071764)
Supplement: Supplementary file 1 [file cancers-14-01764-s001.zip › SuppleTable S4.pdf]

Supplementary Table S4. Comparison of allele fraction of mutated genes in bronchoalveolar lavage (BAL) fluid.

Factor	Variables	AF (%)	*p* value
Smoking history	Smoker	18.4 ± 6.4	0.013
	Non-smoker	4.6 ± 14.7	
Tumor location	peripheral	14.8 ± 14.6	0.503
	middle, central	10.5 ± 12.8	
Tumor size	≤ 20 mm	9.4 ± 13.0	0.190
	> 20 mm	16.8 ± 14.3	
Histology	adenocarcinoma	9.2 ± 13.0	0.020
	others	22.1 ± 12.3	
Lymph node metastasis	N0	12.1 ± 11.9	0.205
	N1, 2	22.0 ± 23.3	
Stage	I	12.2 ± 12.1	0.305
	II, III, IV	19.6 ± 20.9	
Pathological v factor	0	9.0 ± 11.2	0.069
	1	19.0 ± 15.4	
Pathological ly factor	0	12.3 ± 12.1	0.340
	1	19.2 ± 21.3	
STAS	negative	12.8 ± 13.1	0.492
	positive	18.2 ± 20.0	

AF, allele fraction; STAS, spread through air spaces.
